# Chronic Inhibition of Mitochondrial Dihydrolipoamide Dehydrogenase (DLDH) as an Approach to Managing Diabetic Oxidative Stress

**DOI:** 10.3390/antiox8020032

**Published:** 2019-02-02

**Authors:** Xiaojuan Yang, Jing Song, Liang-Jun Yan

**Affiliations:** Department of Pharmaceutical Sciences, UNT System College of Pharmacy, University of North Texas Health Science Center, Fort Worth, TX 76107, USA; yxj2011bs@126.com (X.Y.); sj4933749@sxmu.edu.cn (J.S.)

**Keywords:** diabetes mellitus, dihydrolipoamide dehydrogenase, mitochondria, oxidative stress, reactive oxygen species

## Abstract

Mitochondrial dihydrolipoamide dehydrogenase (DLDH) is a redox enzyme involved in decarboxylation of pyruvate to form acetyl-CoA during the cascade of glucose metabolism and mitochondrial adenine triphosphate (ATP) production. Depending on physiological or pathophysiological conditions, DLDH can either enhance or attenuate the production of reactive oxygen species (ROS) and reactive nitrogen species. Recent research in our laboratory has demonstrated that inhibition of DLDH induced antioxidative responses and could serve as a protective approach against oxidative stress in stroke injury. In this perspective article, we postulated that chronic inhibition of DLDH could also attenuate oxidative stress in type 2 diabetes. We discussed DLDH-involving mitochondrial metabolic pathways and metabolic intermediates that could accumulate upon DLDH inhibition and their corresponding roles in abrogating oxidative stress in diabetes. We also discussed a couple of DLDH inhibitors that could be tested in animal models of type 2 diabetes. It is our belief that DLDH inhibition could be a novel approach to fighting type 2 diabetes.

## 1. Introduction

Adult-onset diabetes mellitus, also known as type 2 diabetes, is caused by insulin resistance followed by β-cell dysfunction [1,2,3]. The hallmark of this metabolic disorder is persistent hyperglycemia in the blood induced by dysregulation of glucose metabolism [4,5,6]. While pathogenesis of type 2 diabetes is multifactorial, oxidative stress has been thought to be the converging event leading to development and progression of type 2 diabetes [7,8,9,10]. As sources of reactive oxygen species-induced oxidative stress are usually endogenous in type 2 diabetes [11,12], managing diabetic oxidative stress by stimulating endogenous antioxidation pathways may provide a novel approach to fighting diabetes.

## 2. Oxidative Stress and Diabetes

When blood glucose is constantly high, there can be a variety of pathophysiological consequences. These include non-enzymatic modifications of proteins by glucose through a process known as glycation [13,14,15], elevated levels of reactive oxygen species (ROS) [15,16] that can cause oxidative damage to proteins, DNA, and lipids [17,18,19,20], and upregulation of metabolic and signaling pathways that can have detrimental effects on glucose metabolism [21,22,23,24,25]. With respect to elevated ROS production, it has been established that nearly all the identified pathways that are upregulated by persistent hyperglycemia can induce or contribute to redox imbalance and ROS production [12,26]. These include the polyol pathway, the protein kinase C activation pathway, the hexosamine pathway, the advanced glycation end products pathway, and the glyceraldehyde autoxidation pathway [8,10]. In addition, upregulation of the poly adenine diphosphate ADP ribosylation pathway and down regulation of the sirtuin 3 pathway have also been implicated in diabetic oxidative stress that accentuates diabetes and its complications [16,27]. Therefore, we believe that stimulation and reinforcement of cellular antioxidation pathways are promising strategies for attenuating diabetic oxidative stress and ameliorating diabetes.

In this article, we postulate that chronic inhibition of mitochondrial dihydrolipomide dehydrogenase (DLDH) can be explored to manage diabetic oxidative stress in diabetic conditions

## 3. Mitochondrial Dihydrolipomide Dehydrogenase (DLDH)

**Figure 1:**
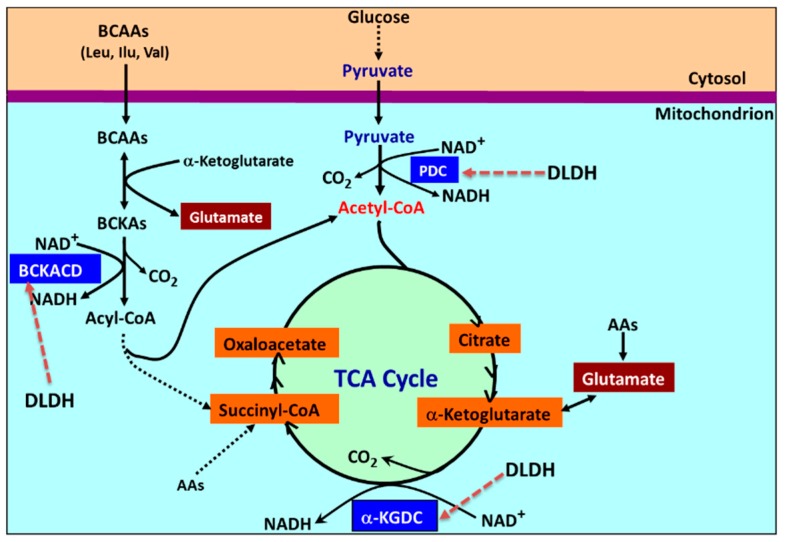
Mitochondrial metabolic pathways involving dihydrolipomide dehydrogenase (DLDH), which include the pyruvate to acetyl-CoA pathway, the α-ketoglutarate to succinyl-CoA pathway, and the branched chain amino acids (leucine, isoleucine, and valine) to acyl-CoA pathway. The glycine cleavage pathway that also involves DLDH is not shown here. DLDH-involved complexes are indicated by dotted red arrows on the figure. BCAA: branched chain amino acids; NAD+: nicotinamide adenine dinucleotide; NADH: reduced form of NAD+; AAs: amino acids; α-KGDC: alpha ketoglutarate dehydrogenase complex; TCA: tricarboxylic acid; BCKA: branched chain keto acid; BCKACD: branched chain alpha keto acid dehydrogenase complex; PDC: pyruvate dehydrogenase complex.

**Figure 2:**
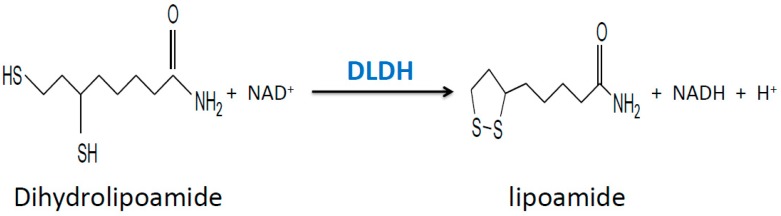
The chemical reaction catalyzed by DLDH. Dihydrolipoamide is oxidized to lipoamide at the expense of NAD+. Hence the DLDH-catalyzed reaction produces NADH that feeds into the electron transport chain in the inner mitochondrial membrane.

Mitochondrial dihydrolipomide dehydrogenase (DLDH) is a flavin adenine dinucleotide (FAD)-containing, nicotinamide adenine dinucleotide (NAD)-dependent disulfide-implicated redox enzyme [28,29,30,31]. DLDH participates in three mitochondrial enzyme complexes, namely pyruvate dehydrogenase complex, α-keto glutarate dehydrogenase complex, and branched chain amino acid dehydrogenase complex (Figure 1). DLDH is also involved in the glycine cleavage system. In the three dehydrogenase complexes, DLDH catalyzes the same reactions that oxidizes dihydrolipoamide to lipoamide (Figure 2) so that the overall enzymatic reactions can continue.

**Figure 3:**
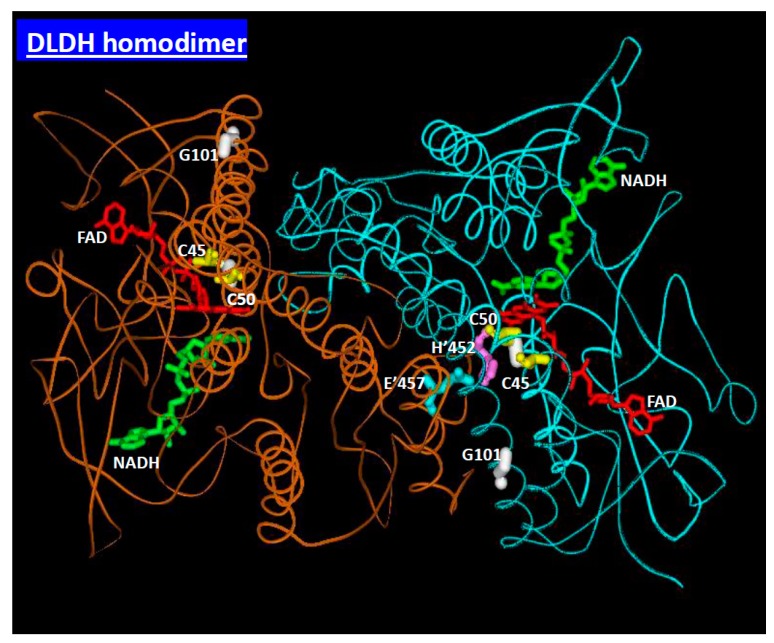
Structure of a DLDH homodimer. Each monomer contributes amino acid residues involved in enzyme activities. Each monomer contains a tightly bound flavin adenine dinucleotide (FAD) molecule, an NAD molecule and two cysteine residues (C45 and C50 in the figure) at the active center. The two cysteines form a disulfide bond (yellow) during catalysis. Electrons from the substrate dihydrolipoamide are eventually transported to NAD via the two cysteine residues and the FAD molecule. This image was created using MDL Chime [41]. Data were derived from the Protein Data Bank (PDB ID: 1ZMC) [42].

DLDH is a multifunctional protein. In rat, the brain and the testis appear to have the highest DLDH activity while the lung gives the lowest DLDH activity [31]. When it exists as a homodimer in the above mentioned dehydrogenase complexes, it is a classical redox-dependent enzyme that converts dihydrolipoamide to lipoamide using two cysteine residues at its active center as a redox relay system (Figure 3). However, the enzyme, when it exists as a monomer, can have a moonlighting function, for example acting as a protease [32]. DLDH can either enhance or attenuate production of reactive oxygen species (ROS), depending on experimental or pathophysiological conditions [29,33,34,35,36,37,38]. In particular, DLDH has two redox-reactive cysteine residues at its active center [39,40] that may scavenge reactive oxygen or reactive nitrogen species, thereby bearing the brunt of oxidative attack and sparing other macromolecules from oxidative damage.

In our recent studies, we have found that DLDH can be inactivated by mitochondrial ROS or by reactive nitrogen species (RNS) [38,43,44]. These studies, on the other hand, would indicate that DLDH could serve as an antioxidant enzyme under stress or pathophysiological conditions. As age-related metabolic stress involves reactive oxygen or nitrogen species [45,46], we reason that enhancing DLDH’s ability to scavenge ROS or RNS may serve as approaches for retardation of development of age related metabolic syndrome including diabetes; and such enhancement may be achieved by chronic inhibition of DLDH in vivo, which should be designed not to pose any toxicity but to lend the benefits of anti-oxidation property of a chronically inhibited DLDH [44]. Below we will discuss what happens when DLDH is chronically inhibited. Please note that we will not cover acute inhibition of DLDH activity, as such an approach may pose toxicity to experimental subjects.

## 4. Accumulation of Dihydrolipoamide

A direct consequence of DLDH inhibition is the blockade of dihydrolipoamide conversion to lipoamide. This would induce accumulation of dihydrolipoamide in the cell [47]. As dihydrolipoamide contains two free sulfhydryl groups, it has a strong antioxidation ability [39,48,49,50,51]. Therefore, accumulation of dihydrolipoamide upon chronic inhibition of DLDH may give cells the ability to eliminate more reactive oxygen or nitrogen species under diabetic conditions.

It should be noted that in mouse studies, loss of 50% DLDH activity does not yield any diseased phenotypes [52,53]. This demonstrates that partial inhibition of DLDH is feasible and does not impair thriving of the DLDH-deficient animals. Nonetheless, benefits resulting from chronic DLDH inhibition in diabetic rodent models have yet to be evaluated.

## 5. Accumulation of Pyruvate

**Figure 4:**
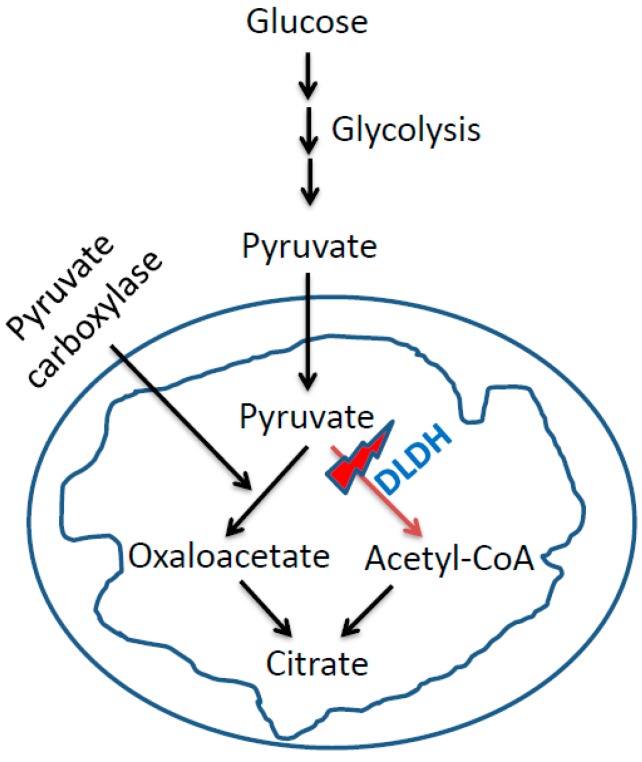
DLDH inhibition could divert pyruvate to the pathway of oxaloacetate formation, which could be further channeled into the anaplerotic pathway.

Chronic inhibition of DLDH may also induce accumulation of pyruvate in a cell (Figure 4). Pyruvate, as has been established, is an antioxidant that could be neuroprotective or cardiac protective [54,55,56,57,58]. Nonetheless, whether pyruvate is accumulated following chronic DLDH inhibition in diabetic subjects needs to be investigated.

Another possibility of pyruvate accumulation is the shift of pyruvate towards oxaloacetate (OAA) production that is catalyzed by pyruvate carboxylase. This reaction is involved in anaplerotic metabolism and production of NADPH [59] that may also be explored to fight oxidative stress in diabetes because NADPH is involved in maintaining cellular redox balance and glutathione metabolism [60,61,62,63].

## 6. DLDH Chronic Inhibition and Activation of Nrf2 Signaling Pathway

**Figure 5:**
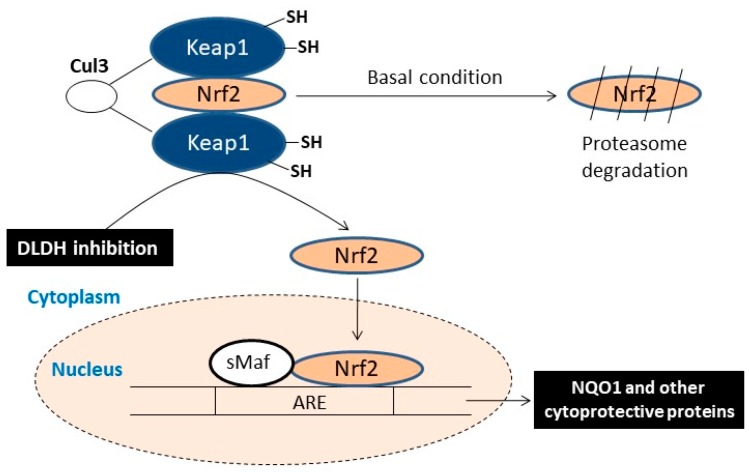
Scheme showing that inhibition of DLDH could activate the Nrf2 signaling pathway, resulting in upregulation of antioxidant enzymes including NAD(P)H quinone dehydrogenase 1 (NQO1) and heme-oxygenase-1 as well as other antioxidant enzymes.

We have found that in stroke neuroprotection studies, inhibition of DLDH by 5-methoxyindole-2-carboxylic acid (MICA) was able to activate the Nrf2 signaling pathway that leads to NAD(P)H quinone dehydrogenase 1 (NQO1) upregulation [44,64]. This pathway was clearly demonstrated in both preconditioning and postconditioning in rat brain by MICA treatment, demonstrating that chronic inhibition of DLDH could be used as a potential approach for targeting oxidative stress in metabolic diseases (as shown in Figure 5). Studies by others also indicate the involvement of DLDH in the activation of the Nrf2 signaling pathways [65,66]. More recently, DLDH inhibition by MICA has also been shown to yield beneficial effects in Alzheimer’s disease [67,68].

## 7. Potential DLDH Inhibitors

Currently, there are two DLDH inhibitors that have been studied. One is MICA [69], and the other is valproic acid (VPA) metabolites. As we have covered MICA in above discussions, we would now discuss VPA metabolites. VPA is an antiepileptic drug [70]. It has been reported that derivatives of VPA such as valproyl-CoA and valproyl-dephospho-CoA can directly inhibit DLDH activity [71], leading to impairment of mitochondrial function reflected by decreased oxygen consumption and decreased ATP synthesis [71]. These derivatives, together with MICA and its derivatives, should also be tested for their antioxidative effects in type 2 diabetes. It should be noted that whether VPA inhibition of DLDH could also lead to activation of the Nrf2 signaling pathway remains unknown. It should also be noted that while arsenite inhibits overall DLDH-catalyzed reaction, arsenite should not be tested in the context of diabetes as this chemical will conjugate with dihydrolipoamide [47,72] rather than the enzyme DLDH itself, leading to less dihydrolipoamide that could be available for antioxidative purposes. It should be pointed out that while arsenite is not suitable to develop in diabetic research, many other natural product(s) or nutrients could be good DLDH inhibitors that will need to be identified for managing diabetic oxidative stress.

## 8. Conclusions

Oxidative stress is a major pathological mechanism of type 2 diabetes. DLDH, as a redox-sensitive enzyme, plays a central role in glucose metabolism. As inhibition of DLDH could induce antioxidative responses and enhance cellular antioxidation capacity in oxidative stress conditions, we propose that chronic DLDH inhibition could serve as a protective approach against oxidative stress in type 2 diabetes, thereby leading to diabetes remission. In this sense, DLDH inhibition may elicit antioxidative effects similar to those induced by treatment with antioxidant-like natural products and/or nutrients.
